# Effect of Alloying on Microstructure and Mechanical Properties of AlCoCrFeNi_2.1_ Eutectic High-Entropy Alloy

**DOI:** 10.3390/ma17184471

**Published:** 2024-09-12

**Authors:** Xue-Yao Tian, Hong-Liang Zhang, Zhi-Sheng Nong, Xue Cui, Ze-Hao Gu, Teng Liu, Hong-Mei Li, Eshkuvat Arzikulov

**Affiliations:** 1 School of Materials Science and Engineering, Shenyang Aerospace University, Shenyang 110136, China; 2 Institute of Physics, Samarkand State University Named after Sharof Rashidov, University Boulevard 15, Samarkand 140104, Uzbekistan

**Keywords:** eutectic high-entropy alloy, alloying, microstructures, mechanical properties, fracture mechanism

## Abstract

In order to explore the effect of alloying on the microstructures and mechanical properties of AlCoCrFeNi_2.1_ eutectic high-entropy alloys (EHEAs), 0.1, 0.2, and 0.3 at.% V, Mo, and B were added to the AlCoCrFeNi_2.1_ alloy in this work. The effects of the elements and contents on the phase composition, microstructures, mechanical properties, and fracture mechanism were investigated. The results showed that the crystal structures of the AlCoCrFeNi_2.1_ EHEAs remained unchanged, and the alloys were still composed of FCC and BCC structures, whose content varied with the addition of alloying elements. After alloying, the aggregation of Co, Cr, Al, and Ni elements remained unchanged, and the V and Mo were distributed in both dendritic and interdendritic phases. The tensile strengths of the alloys all exceeded 1000 MPa when the V and Mo elements were added, and the Mo0.2 alloy had the highest tensile strength, of 1346.3 MPa, and fracture elongation, of 24.6%. The alloys with the addition of V and Mo elements showed a mixed ductile and brittle fracture, while the B-containing alloy presented a cleavage fracture. The fracture mechanism of Mo0.2 alloy is mainly crack propagation in the BCC lamellae, and the FCC dendritic lamellae exhibit the characteristics of plastic deformation.

## 1. Introduction

Due to their unique solid solution structures, high-entropy alloys (HEAs) typically exhibit various excellent properties, such as high strength/hardness [1,2,3,4], high wear resistance [5], outstanding temperature strength [6], strong structural stability [7], good corrosion resistance [8,9], and oxidation resistance [10] Generally speaking, HEAs can be divided into face-centered cubic (FCC) HEAs, body-centered cubic (BCC) HEAs, FCC + BCC two-phase HEAs, etc., from the perspective of phase structure. The high-entropy alloy (HEA) with a single FCC structure has high ductility but low hardness. For example, the CoCrFeMnNi alloy has a room temperature fracture elongation of 52%, but a tensile strength of only 563 MPa [11], and the Fe_40_Mn_40_Co_10_Cr_10_ alloy has a tensile ductility of 58%, but a yield strength of only 240 MPa [12]. Although HEA with a single BCC structure has high strength and hardness, it also has low ductility. Therefore, an important challenge in the engineering application of high-entropy alloys is how to obtain high strength and high ductility at the same time. A potential solution to the problem lies in the development of eutectic high-entropy alloys (EHEAs), which are novel kinds of HEA, with significant advantages. Their composite structures, with FCC and BCC solid solutions, retain both the superior casting performance of eutectic alloys and the excellent mechanical qualities of HEAs.

Lu [13] combined the advantages of FCC and BCC high-entropy alloys, and designed a representative dual-phase eutectic high-entropy alloy AlCoCrFeNi_2.1_ based on the point ratio of eutectic components, which achieves an excellent match of high strength and good ductility. The X-ray diffraction (XRD) analysis showed that the alloy is composed of two phases, FCC and B2 structures, while the tensile strength is 944 MPa and the fracture elongation is 25.6%. Ting X et al. [14] observed scattered nanocrystalline precipitates with BCC structures in the B2 phase and spherical or ellipsoidal L1_2_ precipitates in the FCC phase in an AlCoCrFeNi_2.1_ alloy. The results showed a high level of consistency between the L1_2_ precipitate and its FCC matrix, as well as between the BCC precipitate and its B2 matrix. In order to explore the excellent strength and toughness of AlCoCrFeNi_2.1_ alloy, Gao X et al. [15] found that AlCoCrFeNi_2.1_ alloy has staggered microstructures, which include three-dimensional regular FCC (L12)/BCC (B2) composite structures and two-dimensional lamellar structures. Two different fracture mechanisms were identified in this alloy: ductile fracture in the FCC (L12) phase and brittle fracture in the BCC (B2) phase. The AlCoCrFeNi_2.1_ alloy was treated by low-temperature rolling and annealing processes, and new hierarchical microstructures formed [16]. These results showed that the yield strength (1437 ± 26 MPa), tensile strength (1562 ± 33 MPa), and elongation (14 ± 1%) of the AlCoCrFeNi_2.1_ alloy after annealing at low temperature were significantly improved compared with the as-cast alloy.

Eutectic high-entropy alloys exhibit an excellent balance of toughness and strength at room temperature. However, there is still an immediate requirement to design structural components with more strength and ductility due to the increasing technical requirements and harsher operating environments in industrial applications. The demand for stronger and more ductile structural elements has increased as airplane components have become more demanding and technical standards have advanced. Therefore, it is of significance to develop a new eutectic high-entropy alloy with superior properties based on AlCoCrFeNi_2.1_.

Because AlCoCrFeNi_2.1_ EHEAs present excellent properties, far beyond those of single-phase high entropy-alloys, they have been widely studied by scholars as new types of HEA. However, the current treatment methods for improving strength and toughness are relatively complicated, and how to further improve the properties is the key to developing these alloys. Alloying is a simple method that can effectively improve the mechanical properties and expand the application range of alloys. The majority of the additional elements in previous studies were metals, while few studies were performed on how high-melting-point elements and non-metallic elements affect the mechanical characteristics. Therefore, the aim of this work is to improve the strength and ductility of AlCoCrFeNi_2.1_ EHEA alloys by the alloying method. The influence of alloying components and their content on the microstructures and mechanical properties of the alloy were investigated by adding the refractory metal elements V and Mo and the non-metallic element B to the AlCoCrFeNi_2.1_ alloy. The results will provide theoretical guidance for the development of new high-strength and high-toughness eutectic high-entropy alloys.

## 2. Materials and Methods

**Figure 1:**
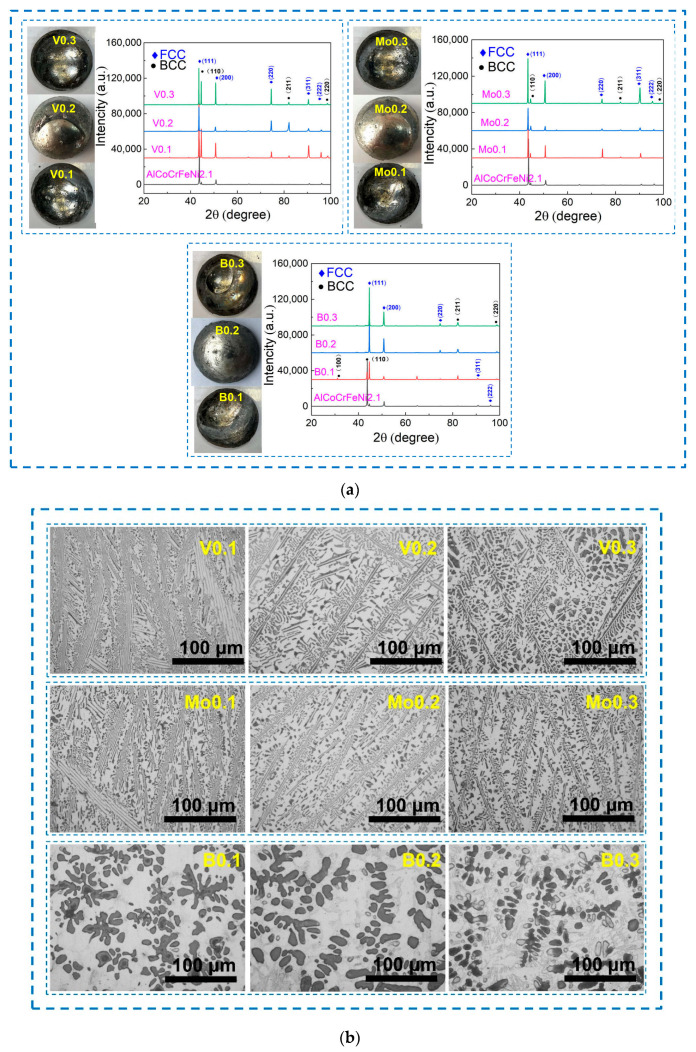
XRD diffractions patterns (**a**) and microstructures (**b**) of AlCoCrFeNi_2.1_X_y_ (X = V, Mo, B; y = 0.1, 0.2, 0.3) EHEAs.

In the experiment, raw materials with purity greater than 99.5% were selected, and the WK-II non-consumable vacuum arc melting furnace (SKY TECHNOLOGY DEVELOPMENT CO., LTD., Shenyang, China) was used to prepare the as-cast ingots in an argon-filled environment. After 4 iterations of repeated melting, the ingots weighing about 80 g were obtained. The ingots were heated at 200 °C for 3 h to remove the effects of residual stress. For ease of representation in this work, AlCoCrFeNi_2.1_X_y_ (X = V, Mo, B; y = 0.1, 0.2, 0.3) EHEAs are denoted as V0.1, V0.2, V0.3, Mo0.1, Mo0.2, Mo0.3, B0.1, B0.2, and B0.3, respectively. According to the morphologies of the alloy ingots presented in Figure 1a, the surfaces of the alloy ingots are uniform and there are no obvious defects, indicating that the target alloys were successfully prepared. The phase compositions of the alloy were analyzed by X-ray diffractometer (Smart Lab 9k, Rigaku Corporation, Japan). The scanning range was 20°~100°, and the scanning speed was 5°/s. In order to observe the microstructures, the samples of 10 mm × 10 mm × 2 mm were cut from the ingots by the electric spark wire-cutting machine. After polishing the sample, the corrosion liquid (hydrofluoric acid, nitric acid, and water = 1:6:7 in volume fraction) was used to etch the alloy surface. In order to prepare the sample characterized by electron backscatter diffraction (EBSD), the residual stress on the surface of the polished surface was removed by electrolytic polishing, and the etching solution was perchlorate alcohol with a concentration of 10% (volume fraction). Electrolytic polishing was carried out at a corrosion potential of 30 V at room temperature, and the corrosion time was 10 s. The microstructures and element distribution of the alloys were observed by the GX71 optical microscope (OLYMPUS, Japan), ZEISS-14-type scanning electron microscope (SEM, ZEISS, Germany), and accompanying energy dispersive spectrometer (EDS). The MHV-1000Z digital micro-hardness tester (Shenyang Weilian Technology Co., LTD., Shenyang, China) and WEW-200 universal testing machine (Shandong Kece Test Technology Co., LTD., Jinan, China) were used to further explore the hardness and tensile properties of the alloys. The loads selected for the hardness experiment were 100 g, 200 g, 300 g, 500 g, and 1000 g, and the sample was kept for 15 s under this load. The size of the standard distance section of the tensile test sample was 8 mm × 2 mm × 2 mm, and the tensile rate was set as 10^−3^ s^−1^.

## 3. Results and Discussion

### 3.1. Microstructures and Phase Compositions

Figure 1a shows the XRD diffraction patterns of the AlCoCrFeNi_2.1_X_y_ (X = V, Mo, B; y = 0.1, 0.2, 0.3) EHEAs. The AlCoCrFeNi_2.1_X_y_ alloys are still composed of two-phase solid solutions with BCC and FCC structures, which are consistent with the crystal structures of the AlCoCrFeNi_2.1_ alloy after the addition of non-metallic element B and high-melting-point metal elements V and Mo. The location of the main strongest peak for the AlCoCrFeNi_2.1_Vy and AlCoCrFeNi_2.1_Moy alloys decreases and then increases with the addition of V and Mo contents, whereas the position movement of this diffraction peak shows the opposite change with the increase in the B element. This implies that the proportion of FCC phase in the AlCoCrFeNi_2.1_X_y_ alloys decreases and then increases when the contents of V and Mo are raised from 0.1 at.% to 0.3 at.%, and that the proportion of FCC phase in AlCoCrFeNi_2.1_By alloy increases and then decreases with the addition of B.

Figure 1b shows the microstructures of the AlCoCrFeNi_2.1_X_y_ EHEAs. Typical eutectic structures were observed in the AlCoCrFeNi_2.1_X_y_ alloy when the high-melting-point metal elements V and Mo were added. The lamellar structures were formed by the alternating distribution of the FCC and BCC phases, and the number of coarse dendrites also increases with the addition of more high-melting-point metal elements. The dendritic structures of the V0.2 and Mo0.2 alloys are greater than those of the V0.1, V0.3, Mo0.1, and Mo0.3 alloys. This is consistent with the results for the XRD diffraction peak strength in Figure 1a. Typical eutectic lamellar structures are not formed after the addition of the B element in the AlCoCrFeNi_2.1_ alloy, and the grain sizes of the AlCoCrFeNi_2.1_By alloys are obviously larger than those of the alloys with the addition of the high-melting-point metal elements V and Mo. The grain sizes of the AlCoCrFeNi_2.1_By alloys gradually decrease as the content of B increases from 0.1 at.% to 0.3 at.%, and the proportion of the dendrite region also gradually decreases. Because the dendrite region is FCC phase [13], this indicates that the proportion of FCC phase in the AlCoCrFeNi_2.1_By alloy decreases with the increase in the content of non-metallic element B, which is consistent with the XRD results.

**Figure 2:**
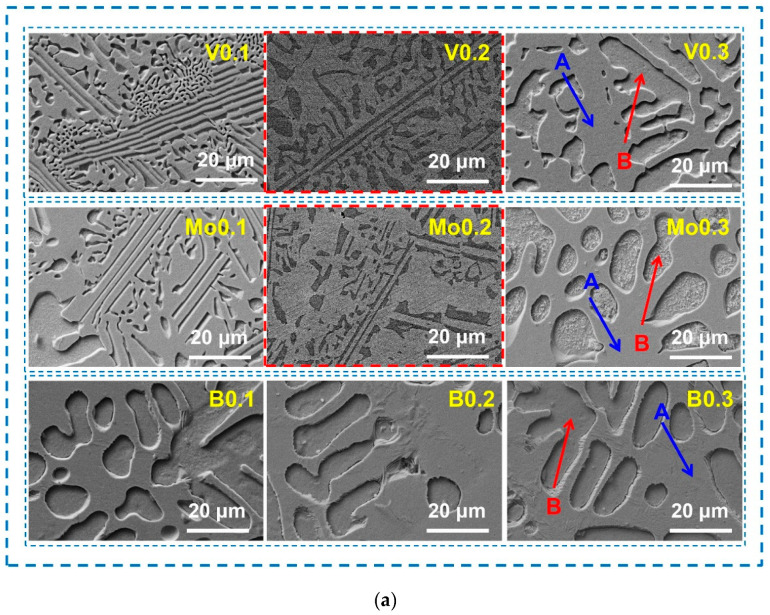

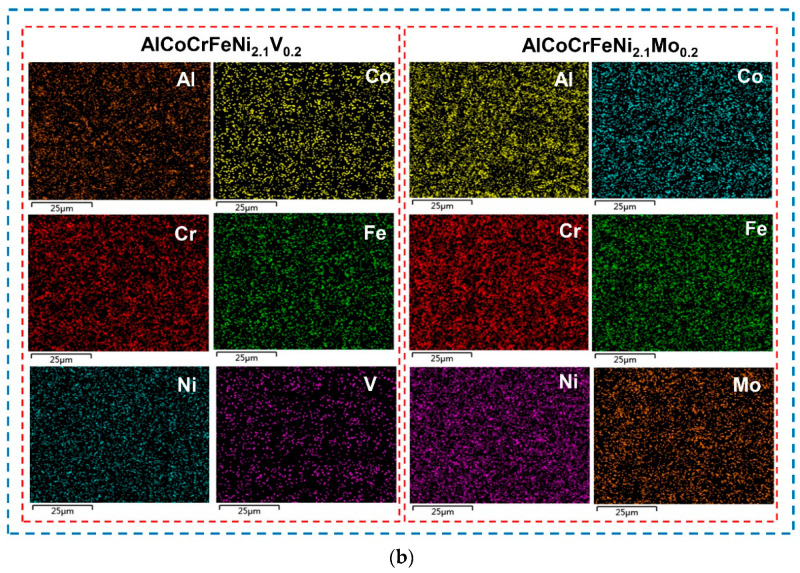
SEM images of AlCoCrFeNi_2.1_X_y_ (X = V, Mo, B; y = 0.1, 0.2, 0.3) EHEAs (**a**), and element distribution of V0.2 and Mo0.2 alloys (**b**).

Figure 2a shows the SEM microstructures of the AlCoCrFeNi_2.1_X_y_ (X = V, Mo, B; y = 0.1, 0.2, 0.3) EHEAs. Typical eutectic structures consisting of a dendritic area (region A) and an interdendritic region (region B) are found in these alloys. In eutectic high-entropy alloys, the dendrite region is usually FCC solid solution and the interdendrite region is BCC solid solution [13]. The element distribution of the AlCoCrFeNi_2.1_X_y_ EHEAs listed in Table 1 indicates that the Co, Cr, and Fe are primarily enriched in the FCC phase (region A) of the V-containing alloys, while the Al and Ni are enriched in the BCC phase (region B). The map scanning analysis of V0.2 (Figure 2b) shows that the Co and Fe elements tend to congregate while the V elements have a tendency to disperse in the dendritic region. The element distribution of the Mo0.1 and Mo0.2 alloys with the high-melting-point Mo is the same as that in the V-containing alloys. However, when the content of Mo rises to 0.3 at.%, a significant amount of Cr and Mo elements congregate in the BCC phase (region B). Figure 2b shows that the aggregation of Al elements is obvious, and that the Mo and V elements tend to be dispersed in the dendritic region. At the same time, a large number of fine precipitates were observed on the surface of the BCC phase (region B) of the Mo0.3 alloy. This may have been the formation of the intermetallic compounds composed of Cr and Mo elements. The element distribution of the B0.3 and B0.2 alloys with the addition of non-metallic element B was close to the nominal composition and was more uniform. A large number of Cr elements were identified in the BCC phase (region B) of the B0.1 alloy. However, due to the tiny atomic radius of B, it is difficult to detect by the EDS method.

### 3.2. Mechanical Properties

**Figure 3:**
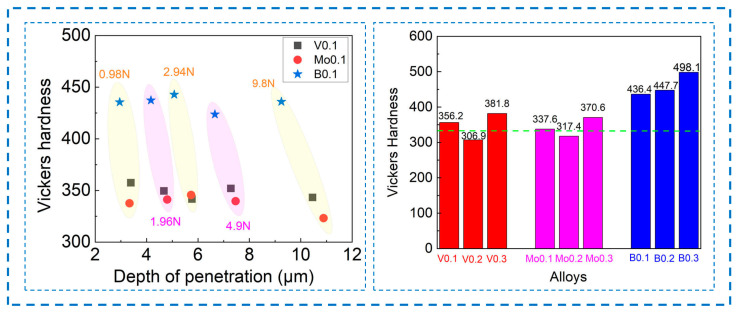
Hardness profile corresponding to the depth of penetration and hardness of AlCoCrFeNi_2.1_X_y_ (X = V, Mo, B; y = 0.1, 0.2, 0.3) alloys.

Figure 3 shows the hardness of the AlCoCrFeNi_2.1_X_y_ (X = V, Mo, B; y = 0.1, 0.2, 0.3) eutectic high-entropy alloys. Firstly, the relationship between the alloy hardness and the depth of penetration of the micro-indenter under different loads was tested before the hardness testing of the different samples. The V0.1, Mo0.1, and B0.1 alloys were chosen as the test samples. It is easy to see that the depth of penetration increases with the increase in the loads, while the hardness of the alloy is basically maintained in a certain range. The hardness values of the V0.1, Mo0.1, and B0.1 alloys were about HV350, HV330, and HV430, respectively. This shows that the method of the hardness test in this work is reasonable and effective. In order to ensure the accuracy of the data, the hardness test in this work was carried out under the load of 500 g. Furthermore, the green center line in the right of the figure is the hardness of the base AlCoCrFeNi_2.1_ alloy. The V0.2 and Mo0.2 alloys have lower hardness values, while the other alloys have higher hardness values than the AlCoCrFeNi_2.1_ alloy. The hardness of the alloy first decreases with the addition of the high-melting-point metal elements V and Mo, and then increases as the content of added elements increases. The alloy reaches its maximum hardness when 0.3 at.% is added. The V0.3 and Mo0.3 alloys have hardness values of HV370.6 and HV381.8, respectively. The hardness values of different phases in alloys can provide evidence for the variation of alloy hardness. Due to the small size of the phases, the dendrite and interdendrite regions (see Figure 1b) of the alloy were tested by a 100 g load in this work. The test results show that the microhardness values of the dendrites are about HV430, HV390, and HV480 when the V, Mo and B are added to the AlCoCrFeNi_2.1_ alloy, respectively. Regarding the interdendrite region, the hardness values are HV300, HV260, and HV370 in these three alloys, respectively. This indicates that the hardness of the interdendritic phase with the FCC structure is lower than that with BCC structure. In addition, when the B element is added, the hardness of both phases across the alloy is significantly increased. This may be related to the formation of different morphologies in the alloys. Since the FCC phase is a soft phase, an increase in its content will somewhat lessen the hardness. It can be seen that the increase in the FCC phase contents in the V0.2 and Mo0.2 alloys results in a decrease in the hardness. The addition of nonmetallic element B has the most noticeable effect on the hardness. When 0.1 at.% of B is added, the hardness of the B0.1 alloy reaches HV436.4. As the element B content increases, the hardness of the alloys increases. The hardness of the B0.3 alloy is HV498.1, which is 48% higher than that of the base alloy. When B element is added, the hardness of the AlCoCrFeNi_2.1_ alloy is significantly increased compared with the addition of the high-melting-point elements V and Mo. On the one hand, this is because when B element is added, the microstructures of alloy change from typical lamellar eutectic morphologies to a petal-like structure (see Figure 1b), in which the petal-like dendrites with BCC structures are coarser, which is conducive to the improvement of the hardness of alloy. Meanwhile, with the increase in the B content, the proportion of BCC phase also increases, and the increase in the harder BCC phase leads to an increase in hardness. On the other hand, unlike the alloys with the addition of V and Mo, the grain refinement is obvious in the alloys with the increase in the content of B. The strengthening effect brought about by grain refinement is also the main reason for the increase in the hardness of these alloys. In conclusion, the addition of high-melting-point metal and nonmetallic elements has a direct impact on the hardness of the AlCoCrFeNi_2.1_ alloy, with nonmetallic elements having the greatest effect.

**Figure 4:**
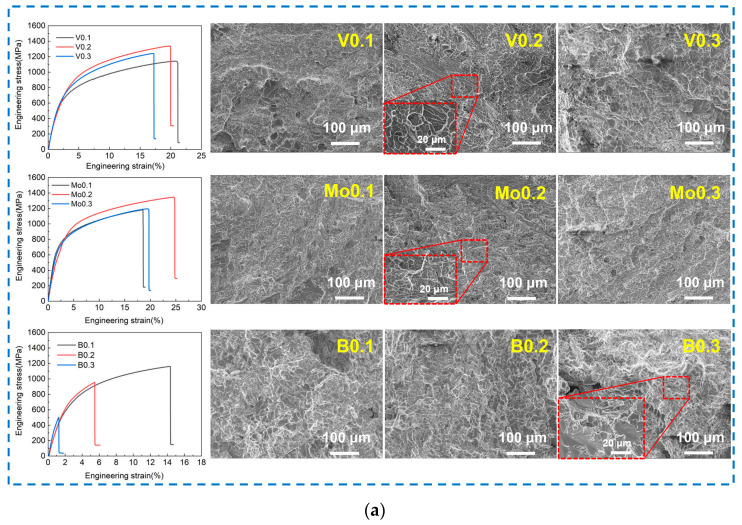

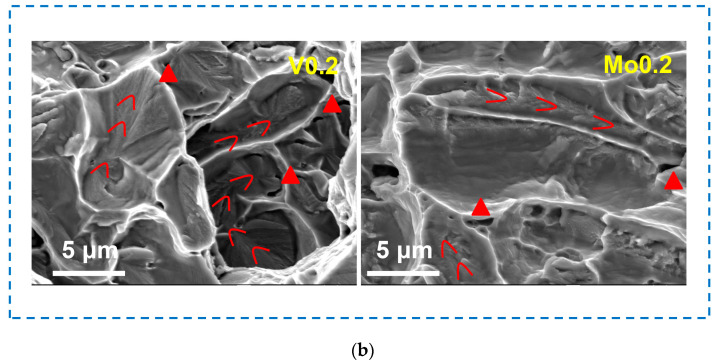
Stress–strain curves and fracture topography of AlCoCrFeNi_2.1_X_y_ (X = V, Mo, B; y = 0.1, 0.2, 0.3) alloys (**a**) and fracture mechanisms of V0.2 and Mo0.2 alloys (**b**).

Figure 4a shows the stress–strain curves and corresponding fracture morphologies of the AlCoCrFeNi_2.1_X_y_ (X = V, Mo, B; y = 0.1, 0.2, 0.3) EHEAs. The tensile strength of the alloys increases and then decreases with the increase in the high-melting-point metals V and Mo, which results from the change in the ratio of the FCC phase to the BCC phase in these alloys. The mechanical properties of the AlCoCrFeNi_2.1_X_y_ alloy are more affected by changes in the B contents compared with the V and Mo. The tensile strength of the AlCoCrFeNi_2.1_By alloy decreases significantly with increasing B elements, and the best mechanical properties were found in the B0.1 alloy. The tensile property of the Mo0.2 alloy is superior to that of the other two alloys with different contents. The tensile strength of the AlCoCrFeNi_2.1_ alloy is greatly increased after adding 0.2 at.% Mo, while the alloy still retains a fracture elongation of more than 24%, as listed in Table 2. The Mo0.2 alloy presents the maximum strength and plasticity when compared to other types of eutectic high-entropy alloy. Its tensile strength is enhanced by 3.6~34%, and its fracture elongation is raised by 0.9~2.9 times.

The SEM images of the fracture in Figure 4a indicate that the fracture mechanism of the alloys containing high-melting-point elements V and Mo is a mixed fracture of toughness and brittleness. Different shapes, sizes, and depths of dimples around bright white tearing edges can be seen in the fracture morphologies. These morphologies are attributed to the ductile transgranular fracture of the FCC phase. In addition, the morphologies of river patterns can be seen, which resulted from the fracture of the BCC phase. The bright and smooth fractures are distributed in the alloys containing non-metallic element B, and the ladder along the direction of the crack growth can be observed. The B0.3 alloy has the highest cleavage step size, which also accounts for its comparatively low tensile property.

The mixed fracture modes of the V0.2 and Mo0.2 alloys can be determined by comparing the fracture morphology in Figure 4b. The crack nucleates at one end of the interdendritic phase of the BCC structure and propagates through the lamella to the other end by leaving a fishbone crack mark with radial stripes (marked with solid red lines) in the BCC lamella. However, the dendritic lamellae of the FCC structure can be necked into lines with no pits (bright lines), exhibiting relatively complete plastic deformation. The radial direction of the fringes in the BCC phase follows the direction of crack propagation, and all the crack sources (represented by triangles) are located on the phase boundary on the short axis. This is due to the fact that stress can easily accumulate on the short axis.

**Figure 5:**
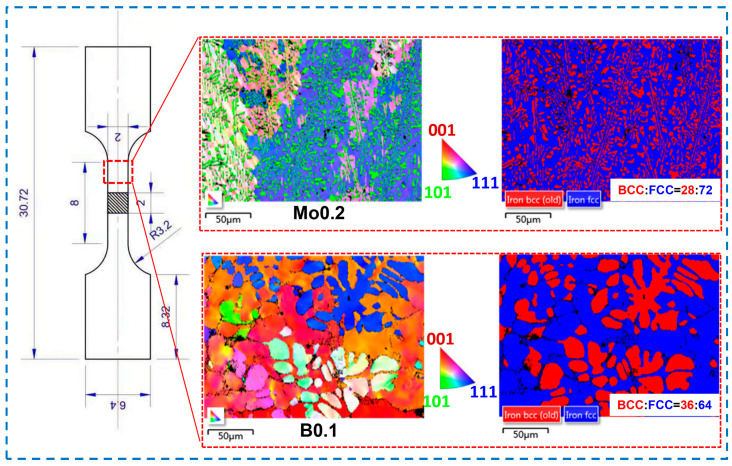
Distribution of grain orientation and phase composition of Mo0.2 and B0.1 alloys after tensile test.

Figure 5 shows the phase composition distribution and grain orientation of Mo0.2 and B0.1 alloys that have better tensile characteristics. The sampling point is close to the fracture surface. In the orientation distribution diagram, different colors stand for various orientation distributions. The <001> orientation is represented by red, the <101> orientation by green, and the <111> orientation by blue. It can be seen that the grain orientation distribution of the B0.1 alloy becomes more disordered after tensile fracture, resulting in a reduction in the toughness, while the orientation between neighboring grains in the Mo0.2 alloy is more consistent, which is because the fine microstructures facilitate the coordination of deformation easier. This improved coordination deformation ability makes the toughness of the alloy increase significantly. The phase composition maps of the alloys show that the volume ratio of BCC and FCC in Mo0.2 is 28:72, while the volume ratio of these two phases in B0.1 is 36:64. This indicates that the FCC phase proportion in this EHEA will decrease with the addition of B elements. The proportion of FCC phase in Mo0.2 alloy is higher than that in B0.1 alloy, and this large amount of FCC phase gives Mo0.2 alloy better plasticity than B0.1 alloy. The BCC phase proportion of B0.1 alloy is higher than that of Mo0.2 alloy, and this large amount of BCC phases gives B0.1 alloy better hardness than Mo0.2 alloy.

## 4. Conclusions

The effects of element contents on the microstructures and mechanical properties of AlCoCrFeNi_2.1_ EHEAs were investigated in this work by using the alloying method. The main conclusions are as follows:

(1) The AlCoCrFeNi_2.1_ alloy is still composed of two solid solutions with FCC and BCC structures after V, Mo, and B are added. The content of the FCC phase decreases and then increases when high-melting-point elements V and Mo are added, while the content increases and then decreases when non-metallic element B is added.
(2) Typical microstructures composed of dendrites and interdendrites were observed in the AlCoCrFeNi_2.1_X_y_ (X = V, Mo, B; y = 0.1, 0.2, 0.3) alloys. Co, Cr, and Fe elements are enriched in the FCC phase, Al and Ni elements are enriched in the BCC phase. The high-melting-point elements V and Mo are distributed in both the FCC phase and the BCC phase.
(3) The tensile strength of the alloy increases and then decreases with the increase in the content of V and Mo elements, while the strength of the alloy decreases significantly with B added. The Mo0.2 alloy achieves a balance of strength and plasticity, and the tensile strength and elongation are 1346.3 MPa and 24.6%, respectively.
(4) The fracture mechanism of the alloys with the addition of V and Mo is a tough–brittle mixed fracture, while the alloy with the addition of B is a cleavage fracture. The propagation of cracks in BCC lamellae was observed in the V0.2 and Mo0.2 alloys, and the FCC dendritic lamellae exhibited perfect plastic deformation.

## Figures and Tables

**Table 1:** Element distribution of AlCoCrFeNi_2.1_X_y_ (X = V, Mo, B; y = 0.1, 0.2, 0.3) EHEAs (at.%).

Alloy	Regions	Al	Co	Cr	Fe	Ni	V/Mo/B
V0.1	Nominal	16.13	16.13	16.13	16.13	33.87	1.61
	A	10.06	20.00	21.49	19.26	27.72	1.46
	B	21.35	15.14	12.03	13.03	37.26	1.19
V0.2	Nominal	15.87	15.87	15.87	15.87	33.33	3.17
	A	10.59	19.5	19.08	18.57	29.28	2.99
	B	27.87	13.44	8.76	9.96	37.4	2.57
V0.3	Nominal	15.63	15.63	15.63	15.63	32.81	4.69
	A	11.20	17.83	18.27	17.18	30.22	5.30
	B	22.15	14.92	11.57	12.83	34.27	4.26
Mo0.1	Nominal	16.13	16.13	16.13	16.13	33.87	1.61
	A	11.47	18.79	18.51	17.02	32.96	1.24
	B	23.50	15.50	12.81	12.17	35.41	0.62
Mo0.2	Nominal	15.87	15.87	15.87	15.87	33.33	3.17
	A	11.05	17.64	18.68	18.36	30.46	3.82
	B	28.11	12.09	10.63	10.74	36.74	1.69
Mo0.3	Nominal	15.63	15.63	15.63	15.63	32.81	4.69
	A	14.26	16.55	18.02	14.37	33.81	2.99
	B	24.03	20.50	14.89	17.33	14.34	8.91
B0.1	Nominal	16.13	16.13	16.13	16.13	33.87	1.61
	A	12.86	17.92	17.72	19.16	32.33	0
	B	14.08	15.72	32.73	14.88	22.58	0
B0.2	Nominal	15.87	15.87	15.87	15.87	33.33	3.17
	A	12.94	18.33	18.69	18.90	31.14	0
	B	17.83	14.81	22.83	14.10	30.43	0
B0.3	Nominal	15.63	15.63	15.63	15.63	32.81	4.69
	A	22.34	12.98	19.84	10.94	33.90	0
	B	14.48	19.57	20.50	17.37	28.09	0

**Table 2:** Mechanical properties of eutectic high-entropy alloys.

Alloys	Phase Composition	Tensile Strength (MPa)	Elongation at Break (%)
Mo0.2	FCC + BCC	1346.3	24.6
B0.1	FCC + BCC	1160.9	14.3
AlCoCrFeNi_2.1_ [13]	FCC + BCC	944	25.6
AlCrFeNi_3_ [17]	FCC + BCC	1200	10.1
Al_0.9_CoFeNi_2_ [18]	FCC + BCC	1005	6.2
Cu_40_Al_20_Ti_20_V_20_ [19]	FCC + BCC	1300	6.3
Ti_0.15_AlCoCrFeNi_2.1_ [20]	FCC + BCC	1253	12.9

## Data Availability

The data presented in this study are available on request from the corresponding author.
